# Empathy Impairment in Individuals With Autism Spectrum Conditions From a Multidimensional Perspective: A Meta-Analysis

**DOI:** 10.3389/fpsyg.2019.01902

**Published:** 2019-10-09

**Authors:** Youming Song, Tingting Nie, Wendian Shi, Xudong Zhao, Yongyong Yang

**Affiliations:** Department of Psychology, School of Education, Shanghai Normal University, Shanghai, China

**Keywords:** autism spectrum conditions, empathy, three-component two-level, culture, gender, age, meta-analysis

## Abstract

**Background:** Although empathy has always been considered to be impaired in individuals with autism spectrum conditions (ASCs), the relevant findings have been inconsistent. The present meta-analysis aims to determine which empathy components are impaired and how culture, gender, and age moderate such empathy impairment.

**Methods:** By using “Autism,” “Asperger Syndrome,” “Empathy,” and related Chinese synonyms as keywords, we searched the databases of Weipu, Wanfang, CNKI, Web of Science, Science Direct, SpringerLink, and Elsevier through “subject” and “keyword” searches. We also conducted a manual search according to the references. In total, 51 studies from Eastern and Western countries were included in this meta-analysis, which comprised 144 independent effects, 2,095 individuals with ASCs and 2,869 controls without ASCs. For the retrieved data, Hedge's *g* was taken as the quantitative measure of effect, and CMA V2.0 software was used for publication bias tests (by using Rosenthal's Classic Failsafe-*N* and Egger's methods), heterogeneity tests (by using a *Q*-test, *I*^2^-test, and *H*-test) and a moderating effect test (by using a univariate regression model).

**Results:** The results showed that the empathy impairment evident in individuals with ASCs is component specific; that is, trait-cognitive empathy, trait-empathic concern, state-cognitive empathy, and state-empathic concern are impaired, whereas state-empathic accuracy remains intact, and trait-empathic accuracy is superior to the trait-empathic accuracy in neurotypical individuals. The univariate regression model showed that gender moderates the impairment of the trait-empathic concern, trait-empathic accuracy, and state-cognitive empathy in autistic individuals and that age moderates the impairment of the trait-cognitive empathy, trait-empathic accuracy, state-empathic concern, and state-empathic accuracy in autistic individuals. However, culture does not moderate any empathy components (trait-cognitive empathy, trait-empathic concern, or state-cognitive empathy) involved in the present meta-analysis.

**Conclusions:** These findings contribute to ending the controversy over the empathic integrity of individuals with ASCs and shed some light on future research about the empathy impairment of autistic individuals. More specifically, subsequent studies should distinguish specific empathy components and consider the role of gender and age when demonstrating empathy impairment in individuals with ASCs. Moreover, related studies based on Asian collectivist cultural samples and female samples should be further enriched.

## Introduction

Autism spectrum conditions (ASCs) refer to a class of generalized developmental disorders characterized by social interaction and communication disorders, narrow interests, repetition, and stereotypical behaviors (American Psychiatric Association, 2013). Clinically, ASCs are characterized by high morbidity (Sun et al., 2013), a low cure rate (Gor et al., 2012), an early onset, and lifelong duration (Fulton et al., 2014). Because of these characteristics, ASCs not only cause lifelong obstacles to the development of the patients themselves but also places a considerable economic burden on their families (Sun et al., 2013). Therefore, topics related to ASCs are of increasing concern to the community.

As one of the core features of ASCs, reduced social interaction behavior has been considered to be associated with the impaired empathy of individuals with ASCs (Mul et al., 2018). However, the relevant conclusions are inconsistent (Rueda et al., 2015; Senland and Higgins-D'Alessandro, 2016; Bos and Stokes, 2018). Possible reasons for this inconsistency are as follows. First, as the core component of interpersonal interactions (Bos and Stokes, 2018), empathy is multi-layered and multidimensional (Kern Koegel et al., 2016; Argott et al., 2017; Bos and Stokes, 2018; Foell et al., 2018; Khalil et al., 2018; Zhao et al., 2018), and the empathy deficit of individuals with ASCs may be component specific. Second, as an important social cognitive process (Chen and Liu, 2016), empathy is context dependent (Kennedy and Adolphs, 2012; Chen and Liu, 2016; Powell and Roberts, 2017), and the empathy impairment of autistic individuals may be regulated by other factors. Therefore, it is necessary to systematically review and clarify the research status of empathy and to further explore the empathic integrity of autistic individuals and its potential influencing factors.

### The Concept, Structure, and Measurement of Empathy

The definition of empathy varies greatly among researchers (Cuff et al., 2014; Sivaraman, 2017). For example, Davis (1980) defined empathy as a reaction to the observed experiences of another person, Trimmer et al. (2017) defined empathy as the ability to share and understand the emotions and feelings of others, and Pelligra (2011) defined empathy as the capacity to anticipate and share other people's emotional states. Pavey et al. (2012) further defined empathy as the experience of sympathetic emotions and concern for another person in distress. However, in terms of the psychological content involved in empathy, these definitions of empathy can be divided into two categories. One is a trait, ability, or personality tendency, namely, trait empathy, as described by Decety and Moriguchi (2007), Dziobek et al. (2008), Adler et al. (2015), Trimmer et al. (2017), Bos and Stokes (2018), and Foell et al. (2018), all of whom tend to interpret empathy in their studies as an individual's ability to understand and share the emotions and feelings of other people. The other definition of empathy is an instant psychological state or process, namely, state empathy, as described by Davis (1980), Singer and Lamm (2009), Stocks et al. (2011), and Pavey et al. (2012), all of whom tend to interpret empathy as an interpersonal psychological process or state induced by a specific situation or stimulus.

It should be noted that “trait empathy” and “state empathy” are relative. The main difference between them is that “state empathy” is context dependent (Cuff et al., 2014); namely, it requires the induction of a specific situation and stimulus (Van der Graaff et al., 2016; Zhao et al., 2018) and is a short-term, instant psychological state and process of an individual. In contrast, “trait empathy” is relatively stable across time (Cuff et al., 2014) and does not require the induction of a specific situation or stimulus. This division has also determined that the measurement of “trait empathy” is often achieved through offline, self-reported questionnaires (Zhao et al., 2018), such as the empathy quotient (EQ) (Baron-Cohen and Wheelwright, 2004), the Interpersonal Reactivity Index (IRI) (Davis, 1980) and the Questionnaire on Cognitive and Affective Empathy (QCAE) (Reniers et al., 2011). In contrast, the measurement of “state empathy” is often achieved through some online, performance-based methods (Zhao et al., 2018), such as the eyes test (Baron-Cohen et al., 2001), the Multifaceted Empathy Test (MET) (Dziobek et al., 2008), the empathic embarrassment tasks (Adler et al., 2015), etc. Accordingly, “trait empathy” is similar to a personality trait, which is stable and invariable, and its measurement therefore does not need to be motivated by specific tasks, whereas “state empathy” is a psychological state or process manifested by an individual in specific tasks, and its measurement therefore depends on a specific experimental task. Thus, empathy is a multi-layered concept that includes not only offline, relatively stable “trait empathy” but also online, relatively unstable “state empathy.”

In addition, empathy is also a multidimensional concept. For example, some early researchers tended to divide empathy into cognitive empathy and affective empathy and defined cognitive empathy as the process of accurately recognizing and understanding others' feelings and emotions, whereas affective empathy is defined as emotional resonance or empathic accuracy, that is, the process of sharing other people's feelings (Jones et al., 2010; Decety, 2011; Fan et al., 2011; Lang et al., 2011; Pasalich et al., 2014). Some later researchers further incorporated empathic concern—the specific emotional response to a person who is suffering, including sympathy, compassion, and being moved—into the components of empathy (Stocks et al., 2011; Stellar et al., 2015; Van der Graaff et al., 2016; Zhao et al., 2018) and thus formed the three-component view of empathy. It is worth noting that for a long time, prior work did not make a good distinction between empathic concern and empathic accuracy and instead used “affective empathy” to refer to both of these terms (Oliver et al., 2016). However, there are many differences between these concepts. For example, in interpersonal orientation, empathic concern is more others-oriented (Klimecki et al., 2013; Stellar et al., 2015), whereas empathic accuracy is more self-oriented (Lamm et al., 2011; Pérez-Manrique and Gomila, 2018). In the nature of the emotions produced, empathic accuracy requires more isomorphism between self and others' emotions, whereas empathic concern does not require this isomorphism (Zhao et al., 2018). Accordingly, the three-component view of empathy has been supported by an increasing number of studies. For example, Van der Graaff et al. (2016) used facial electromyography to investigate the relationship among different components of empathy and ultimately found that there was a significant correlation between the three components of empathy. As another example, Ashar et al. (2017) believed that empathy should include the three components of cognitive empathy, empathic care (equivalent to empathic concern), and affective empathy (or empathic distress, which is equivalent to empathic accuracy) and found through brain imaging technology that empathic care and affective empathy have their own independent brain systems and biomarkers.

However, most current explorations on the components of empathy remain at the trait level. For example, many researchers in specific studies further divide trait empathy into cognitive empathy, empathic concern, and empathic accuracy (Dziobek et al., 2008; Fan et al., 2014; Decety, 2015; Senland and Higgins-D'Alessandro, 2016; Van der Graaff et al., 2016; Ashar et al., 2017; Zhao et al., 2018). However, we found only two studies that specifically addressed the division of empathy components at the state level: Van der Graaff et al. (2016) divided state empathy into state-cognitive empathy and state-affective empathy based on specific problem guidance, and Powell and Roberts (2017) further divided state empathy into state-cognitive empathy, state-compassionate empathy (equivalent to state-empathic concern), and state-affective empathy (equivalent to state-empathic accuracy). This dynamic suggests that the components of empathy are likely to be isomorphic at the trait and state levels.

There are still many disagreements regarding the definition of empathy, such as a “cognitive and affective conflict,” “automatic and controlled conflict,” “congruent and incongruent conflict,” “self-other distinction and merging conflict,” and “trait and state conflict” (Cuff et al., 2014). However, these disagreements do not seem to go beyond the existing multidimensional levels and components of empathy and are more about the differences among the specific components of empathy. More precisely, compared with cognitive empathy, empathic concern, and empathic accuracy are both related to the specific emotions involved in the empathic process; thus, they are more reflective of the emotional side of empathy (Oliver et al., 2016). Meanwhile, the external expression of emotional empathy is more controlled by the parasympathetic nervous system (Zhao et al., 2018), and the activation of brain regions involved in emotional empathy is more automatic (Shamay-Tsoory et al., 2009), which means that the production process of empathic concern and empathic accuracy may be less in the control of consciousness. Compared with empathic accuracy, the produced emotions of empathic concern are different from those of the empathy source itself (Zhao et al., 2018), which is conducive to the distinction between self and others. For empathic accuracy, in contrast, emotional responses are consistent with or isomorphic to the source of empathy itself, which is not conducive to the distinction between the self and others (Cuff et al., 2014). For the “trait and state conflict,” from the point of view of existing research, whether it is cognitive empathy or empathic concern and empathic accuracy, all have both a trait side (Dziobek et al., 2008; Senland and Higgins-D'Alessandro, 2016; Zhao et al., 2018) and a state side (Powell and Roberts, 2017).

Although empathy varies between layers and dimensions, this does not mean that these dimensions are independent of one another as, at different layers, many studies have found that trait empathy can positively predict state empathy (Sonnby-Borgström et al., 2003; Rae Westbury and Neumann, 2008; Dimberg and Thunberg, 2012; Van der Graaff et al., 2016). Many studies have even found correlations among different dimensions. For example, Zhao et al. (2018) found that trait-cognitive empathy not only significantly predicted trait-empathic concern but also significantly predicted state-empathic concern together with trait-empathic concern. As another example, Dziobek et al. (2008) and Decety et al. (2015) found that trait-empathic concern was significantly correlated with state-empathic concern and state-empathic accuracy. These findings show that empathy itself is a complex, multilevel and multidimensional concept.

Accordingly, although a large number of valuable achievements have been accomplished in the definition of empathy, previous studies have rarely considered the multilevel and multidimensional nature of empathy itself when defining empathy. This has led to a further narrowing of the concept of empathy; namely, it has confused the concept of empathy with the concept of a certain level or component of empathy. As such, Davis's (1980) definition of empathy is more inclined to state empathy, Trimmer et al. (2017) define empathy more as trait-cognitive empathy and trait-empathic accuracy, and Pavey et al. (2012) define empathy more as a reflection of state-empathic concern. Therefore, based on previous work, we believe that empathy reflects an individual's pre-existing perceptual and reactive tendency toward other people's emotions and feelings and the instant process of cognition and reaction of this tendency. It is a multidimensional construct with three components and two levels. It should be noted that our definition of the concept and structure of empathy is more concerned with the psychological characteristics and cognitive processes of empathic individuals themselves. However, whether this concept and structure can be used to explain empathy in the interpersonal interaction process or empathic aftereffects (e.g., the interactive behavior induced by empathy) still requires further demonstration.

In addition, many terminologies have been used to describe the empathy components in existing studies, for example, the terminologies that describe “cognitive empathy” include “emotion recognition” (Soto and Levenson, 2009), “theory of mind” (Blair, 2005; Schwenck et al., 2012; Rueda et al., 2015), “perspective-taking” (Van der Graaff et al., 2016), “empathic accuracy” (Richter and Kunzmann, 2011), and “cognitive empathy” itself. The terminologies that describe “empathic concern” include “empathic care” (Ashar et al., 2017), “compassionate empathy” (Goetz et al., 2010; Powell and Roberts, 2017), “emotional empathy” (Dziobek et al., 2008), “sympathy” (Richter and Kunzmann, 2011), “emotional concern” (Davis, 1994), and “empathic concern” itself. Finally, the terminologies that describe “empathic accuracy” include “empathic distress” (Ashar et al., 2017), “affective empathy” (Richter and Kunzmann, 2011; Van der Graaff et al., 2016; Powell and Roberts, 2017), “emotion contagion” (Hatfield et al., 2009), “emotional empathy” (Fan et al., 2014), “affective sharing” (Oliver et al., 2016; Zhao et al., 2018), “motor empathy” (Blair, 2005), etc. However, the relevant terminologies are not specific to the specific components of empathy. The same terminology can be used to describe different empathy components in different studies. For example, “empathic accuracy” is similar to “cognitive empathy” in Richter and Kunzmannl's (2011) study but reflects more on the “subject's isomorphic experiences of the protagonist's emotions” in Zaki et al.'s (2009) study. “Emotional empathy” is similar to “empathic concern” in Dziobek et al.'s (2008) study, but it is more similar to “empathic accuracy” in Fan et al.'s (2011) study. Alternatively, it can refer to different empathy components in the same study; for example, “affective empathy” refers to both “empathic concern” and “empathic accuracy” in Senland and Higgins-D'Alessandro's (2016) study. These differences have caused great confusion for scholars. Therefore, according to the empathic structure proposed above, we named the three empathy components at the trait level as trait-cognitive empathy (T-CE), trait-empathic concern (T-EC), and trait-empathic accuracy (T-EA), while we named the three empathy components at the state level as state-cognitive empathy (S-CE), state-empathic concern (S-EC), and state-empathic accuracy (S-EA). We hope that our naming of the components of empathy at different levels will intuitively reflect the subordination of the components of empathy to empathy itself conceptually and, to some extent, put an end to the chaotic situation of the mixing and inter-use of empathic terminologies. The specific definitions and measurements of each component are as follows.

Trait-cognitive empathy refers to an individual's ability and tendency to understand and infer other people's beliefs, intentions, and feelings (Decety and Yoder, 2016). The main measurement indexes are the scores on the cognitive empathy subscale of the EQ (Baron-Cohen and Wheelwright, 2004), IRI (Davis, 1980), QCAE (Reniers et al., 2011), and the total score of the Basic Emotional Empathy Scale (BEES) (Mehrabian, 1996).

Trait-empathic concern refers to the tendency of individuals to respond to the specific emotions of people in pain, including their tendency to experience sympathy, experience compassion and be moved (Stocks et al., 2011). The main measurement indexes include the score of the empathic concern subscale on the IRI (Davis, 1980) and EQ (Baron-Cohen and Wheelwright, 2004).

Trait-empathic accuracy refers to the ability and tendency of individuals to share or become affectively congruent with others' emotional states at least in terms of valence and intensity, which is similar to emotional infection and resonance (Decety and Yoder, 2016; Jordan et al., 2016). Currently, the main measurement indexes are the scores on the subscale of personal distress (PD) of the IRI (Davis, 1980) and the affective empathy subscale of the QCAE (Reniers et al., 2011). The affective empathy subscale of the QCAE questionnaire reflects the ability to be sensitive to and vicariously experience the feelings of other people (Mul et al., 2018), which is more consistent with trait-empathic accuracy. In contrast, the PD subscale of the IRI questionnaire reflects the tendency of individuals to generate self-directed uncomfortable feelings in negative situations (Dziobek et al., 2008; Bellebaum et al., 2014), which seems to initially not fit very well with the definition of trait-empathic accuracy. However, empathy is often directed at the suffering of people in negative situations, and sharing in the negative emotions of others who are suffering is usually experienced as self-focused empathic or personal distress (Lamm et al., 2011; Pérez-Manrique and Gomila, 2018). In addition, a study based on brain imaging also found that empathic distress often shares the same brain regions with the sharing of other people's experiences and emotions (Ashar et al., 2017). Therefore, many studies (e.g., Rueda et al., 2015; Decety and Yoder, 2016; Zhao et al., 2018) tend to use the PD subscale of the IRI questionnaire as a tool to measure trait-empathic accuracy.

State-cognitive empathy reflects the immediate cognitive process of the belief in, intention of and feeling for others (Powell and Roberts, 2017). Its main measurement indexes include the score on the state-cognitive empathy items of the Measure of State Empathy (MSE) (Powell and Roberts, 2017), the cognitive empathy score of subjects on the MET (Dziobek et al., 2008), the empathy score of subjects on empathic embarrassment tasks (Adler et al., 2015), the accuracy of emotional judgement of subjects in the “Reading the Mind in the Eyes” test (RMET) (Baron-Cohen et al., 2001, 2015), subjects' recognition of different emotions in emotional facial processing tasks (Cassidy et al., 2015; Rigby et al., 2018) and subjects' pain scores toward the empathy object in pain empathy experiments (Krach et al., 2015).

State-empathic concern reflects an individual's immediate response to the special emotions of people in pain, whereas state-empathic accuracy reflects an individual's immediate sharing and resonance of the isomorphic emotions of other people (Powell and Roberts, 2017). In many studies, the differences between the two are mainly reflected in the differences in problem orientation. State-empathic concern is more focused on the degree of concern for and attention to the target subject in the empathy stimulus and situation, as measured by the items of compassionate empathy on the MSE (Powell and Roberts, 2017), the EED (Emotional Empathy, Direct) score on the MET (Dziobek et al., 2008), items in the empathy response story (Ding and Song, 2017; Zhao et al., 2018), and other items in experimental tasks that can reflect the individuals' feelings of concern, pity, and sympathy for others. However, state-empathic accuracy is more focused on the degree of the subject's isomorphic experiences of the protagonist's emotions (Powell and Roberts, 2017), such as the affective empathy item in the MSE (Powell and Roberts, 2017), the EEI (Emotional Empathy, Indirect) score on the MET (Dziobek et al., 2008), the pain score for the subject in the pain experiment (Krach et al., 2015; De Coster et al., 2017), and other items in experimental tasks that can reflect an individuals' sharing of others' emotions or feelings.

### Empathy Impairment in ASC Individuals

It is often assumed that individuals with ASCs lack empathy (Baron-Cohen and Wheelwright, 2004; Tavassoli et al., 2018), especially at the trait level (Rigby et al., 2018). However, this “established fact” is challenged if the multiple levels and components of empathy are discussed in detail. Many studies have found that ASC individuals show intact trait-affective empathy (Mazza et al., 2014; Rueda et al., 2015; Senland and Higgins-D'Alessandro, 2016; De Coster et al., 2017; Vyas et al., 2017; Bos and Stokes, 2018; Mul et al., 2018) and impaired trait-cognitive empathy (Mazza et al., 2014; Rueda et al., 2015; Senland and Higgins-D'Alessandro, 2016; De Coster et al., 2017; Vyas et al., 2017; Bos and Stokes, 2018; Mul et al., 2018) when measured separately. In other studies, however, individuals with ASCs exhibited impaired trait-empathic concern (Hirvelä and Helkama, 2011; Adler et al., 2015; Chung et al., 2016) and intact trait-cognitive empathy (Senland and Higgins-D'Alessandro, 2013; Althaus et al., 2015; Chung et al., 2016). Meanwhile, although autistic individuals reported significantly higher empathic accuracy ability than the empathic accuracy ability of neurotypical individuals in most studies (Hirvelä and Helkama, 2011; Senland and Higgins-D'Alessandro, 2013, 2016; Adler et al., 2015; Althaus et al., 2015; De Coster et al., 2017; Murray et al., 2017; Vyas et al., 2017), some ASC individuals had the same empathic accuracy ability as typically developing individuals (Pouw et al., 2013; Chung et al., 2016; Mul et al., 2018; Thaler et al., 2018).

Even at the level of state empathy, there are also many inconsistencies. For example, individuals with ASCs have reported impaired state-cognitive empathy and intact state-affective empathy on the MET task (Dziobek et al., 2008), whereas other individuals with ASCs have reported intact state-cognitive empathy during the task of observing peer pain stimuli and pain pictures (Bird et al., 2010; Schneider et al., 2013). Similarly, other studies have shown that individuals with ASCs have reported intact state-empathic concern and state-empathic accuracy in the task of pain picture observation (Poustka et al., 2010; Bellebaum et al., 2014); in another study, the opposite conclusion was reached (Campbell et al., 2015). Therefore, it is necessary to systematically investigate the specific empathy impairment of individuals with ASCs and the conditional heterogeneity (such as different cultures, genders, and ages) of their empathy impairment through meta-analytic technology. We hypothesize that the impairment of empathy in ASC individuals is component specific and conditionally heterogeneous to some extent—namely, it is regulated by factors such as culture, gender, and age.

### Potential Moderating Variables for Empathy Impairment in ASC Individuals

#### Culture

Many studies have confirmed cultural differences in empathy (Chentsova-Dutton and Tsai, 2010; Cheon et al., 2013). Generally, it is believed that individuals in other-directed collectivistic cultures have better empathy performance than individuals in self-oriented individualistic cultures (Cheon et al., 2013) because an effective empathy process requires not only paying attention to the feelings and psychological states of other people but also restraining the self-centered concept and emotional state (Lin et al., 2010; Zhao et al., 2018). Meanwhile, studies have also found that individuals in Eastern cultures score significantly higher for autistic traits than individuals in Western cultures (Freeth et al., 2013). In addition, studies have also found that Japanese (Wakabayashi et al., 2006), Malaysian and Indian English-speaking students (Freeth et al., 2013) score significantly higher on the Autistic Quotient (AQ) than British students, whereas other researchers hold that the autistic symptoms of ASC children in the United Kingdom and the United States are more severe than those of ASC children in Israel and South Korea (Matson et al., 2011; Mandy et al., 2014). Therefore, it can be inferred that the level of impairment of empathy in ASC individuals may have cultural influences; that is, culture has a certain regulating effect on the impairment of empathy in ASC individuals.

#### Gender

Gender differences in empathy seem to have become a self-evident fact. In particular, both empirical studies, such as Baron-Cohen and Wheelwright's exploration of gender differences in empathy based on the EQ questionnaire (Baron-Cohen and Wheelwright, 2004) and Baron-Cohen et al. analysis of the gender differences in empathy based on the RMET task (Baron-Cohen et al., 2015), and theoretical explorations, such as the empathizing-systemizing (E-S) theory (Baron-Cohen et al., 2005) and social gender theory (Singer and Lamm, 2009; Ding and Song, 2017), seem to support the idea that females have more advantages regarding empathy than males and that these advantages are cross-culturally consistent. Some clinical studies (Schneider et al., 2013; Tavassoli et al., 2018) and brain imaging studies (Frank et al., 2015) based on ASC individuals have also reached the same conclusion.

In terms of the gender differences in the pathological symptoms of ASCs, some studies have also found that female ASC individuals show fewer repetitive and stereotypical behaviors (Sipes et al., 2011; Frazier et al., 2014; Wilson et al., 2016), less social communication impairment (Hartley and Sikora, 2009; Zwaigenbaum et al., 2012) and more intellectual impairment (Fombonne, 2009) and social stimulus attention preference (Chawarska et al., 2016) than male autistic individuals. The scores of autistic traits are also much lower for females than for males (Freeth et al., 2013; Zhao et al., 2018). Therefore, it can be inferred that there may be gender differences in the impairment of empathy in individuals with ASCs.

#### Age

Conclusions about the age effect of empathy have long been controversial. Specifically, some studies have suggested that empathy increases with age (Sze et al., 2012; Peterson, 2014), whereas other studies have come to the opposite conclusion (Phillips et al., 2002; Ding and Lu, 2016); this is especially true of cognitive empathy (Bailey et al., 2008). In addition, some studies have also found that an individual's ability to experience empathic concern and engage in empathic accuracy increase with age, while cognitive empathy decreases (Richter and Kunzmann, 2011). It seems that the age effect of empathy is component specific; that is, different components of empathy may have completely different age effects. Therefore, it is not difficult to understand why studies (Mestre et al., 2004; Bailey and Henry, 2010) that explore the age effect of empathy from a holistic perspective have failed to draw a corresponding conclusion.

The age effects of autism traits or ASCs have also not been consistently studied. Some studies purport that the pathological symptoms of ASCs decrease with age (Tillmann et al., 2018), whereas other studies show the opposite trend (Powell et al., 2017). In addition, we also compared the diagnostic scores of the autism diagnostic observation schedule (ADOS) in two other studies with similar demographic variables notwithstanding large age differences. We found that the diagnostic scores of ASC individuals (*M*_age_ ± *SD* = 20.92 ± 3.31, *M*_ADOSscore_ ± *SD* = 11.88 ± 2.83) reported by McVey et al. (2016) were significantly higher than the diagnostic scores of ASC individuals reported by Silani et al. (2008) (*M*_age_ ± *SD* = 36.90 ± 11.8, *M*_ADOSscore_ ± *SD* = 9.77 ± 2.40), *t* = 2.34. It can be inferred that age may also be an important variable in regulating empathy impairment in ASCs.

Of course, the empathy impairment of ASC individuals may also be affected by other factors. For example, some studies have found that ASC individuals have varying degrees of impairment in intelligence (Spencer et al., 2007), cognitive control (Solomon et al., 2008), implicit learning (Vivanti and Rogers, 2014), and action anticipation (Ganglmayer et al., 2019). Meanwhile, the impairment of intelligence, cognitive control, implicit learning, and action anticipation in ASC individuals can provide some explanation for their impairment in social communication (Lieberman, 2000; Hughes, 2001; Sinha et al., 2014; Foti et al., 2015; Krogh-Jespersen et al., 2018; Bertollo and Yerys, 2019). However, because the reports on these variables in the study of ASC individuals' empathy impairment are incomplete and the measurement tools are inconsistent, we do not further analyse these variables in this research.

## Methods

### Literature Retrieval

The literature published in Chinese databases (Weipu journal database, Wanfang journal database, CNKI) and English databases (Web of Science, Science Direct, SpringerLink, Elsevier) was retrieved, and manual retrieval was also performed according to the references. The Chinese search keywords mainly included “自闭症,” “孤独症,” “自闭特质,” “阿斯伯格综合征” “共情” “移情” “同理心,” etc., whereas the English search words mainly involved “Autism,” “Asperger Syndrome,” “Empathy,” etc. The main advanced search settings were “subject” and “keyword,” and they included the joint retrieval of “autism” and “empathy.” In light of the systematic attention of this study to the empathy impairment of ASC individuals and the relatively few related studies, no specific time of publication was set in the literature retrieval process. The entire literature retrieval process was simultaneously conducted by five people (one person was responsible for the Chinese database, and the other four people were responsible for the English database) and lasted ~27 days.

### Literature Inclusion Criteria

**Figure 1:**
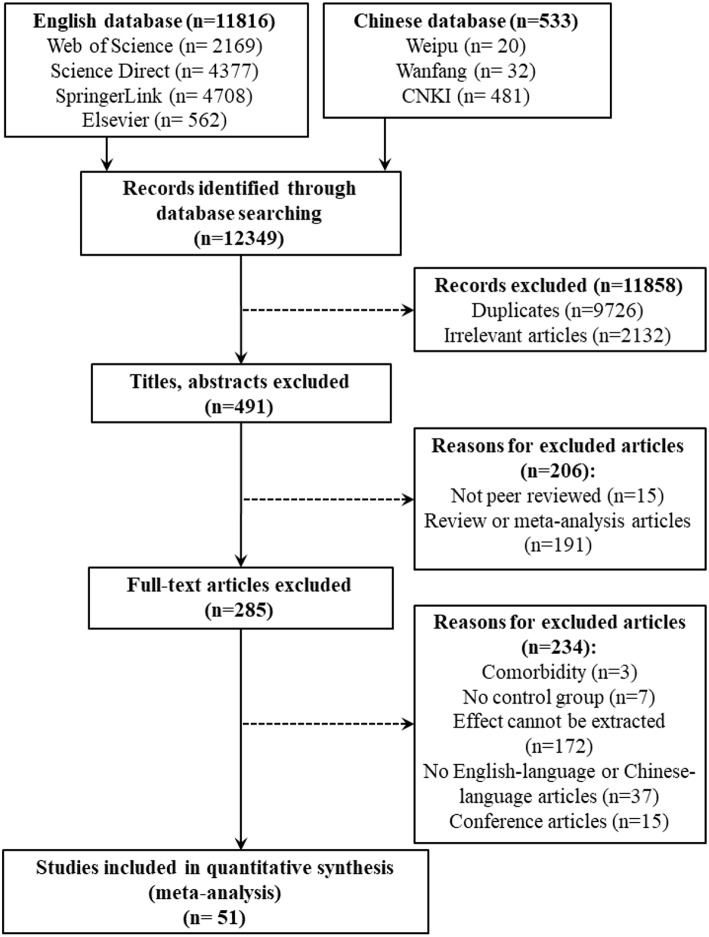
Flow diagram of article inclusion and exclusion.

The retrieved literature was included in the meta-analysis according to the following criteria. First, the included literature needed to be high-quality journal papers that had been published publicly after an anonymous peer review; Master's and doctoral theses were excluded. Second, the included literature had to be empirical or experimental articles; reviews and other non-empirical studies were excluded. Third, the included literature had to comprise both an ASC group and a typically developing (TD) group; studies without a TD group were excluded. Fourth, the included literature had to report the measurement tools, experimental tasks, and specific process of the experiment; otherwise, it was excluded. Fifth, data such as the sample size, mean score of empathy, and standard deviations of the ASC and TD groups had to be reported simultaneously in the included literature; otherwise, the studies were excluded. The specific process of the inclusion and exclusion of the literature is shown in Figure 1.

### Document Coding

First, two independent coders were invited to encode the literature. The content of the coding mainly included cultural background (divided into Eastern or Western culture based on the country's geographical location), age (chronological age), gender [based on the proportion of male subjects and categorized as masculine (≥0.5) or feminine (<0.5)], outcome variables (based on the definition of the empathy components in the Introduction with the studies divided into the T-CE, T-EC, T-EA, S-CE, S-EC, and S-EA categories), group (based on the groups of subjects such as being divided into ASC and TD groups), and the sample sizes of the ASC and TD groups. Second, if multiple independent samples were included in the same study or the same result variable was repeatedly confirmed by different scales, experimental paradigms, and procedures, then weighted mean methods were used to combine the same result variable according to the specific circumstances. Meanwhile, if there were multiple TD groups (or ASC groups) in the same study, the result variables of the multiple TD groups (or ASC groups) were combined into one result variable with the same statistical method, which served as the final result variable of the TD group (or the ASC group) to be compared with the group. Finally, the specific results of the coding of the two coders were compared (coding consistency rate: 93.6%), and the studies with discrepant codings were further verified by a third coder. The final results of the encoding are shown in Table 1.

**Table 1:** Basic information of the articles included in the meta-analysis.

**References**	**Culture**	**Age**	**Gender**	**Empathy tools**	**Group**	**Sample size**	**Education (year)**	**Intelligence**	**ADOS in ASC group**	**AQ**	**Outcome variables*-M* (*SD*)**	**Outcome variables-Hedge's *g***
Adler et al., 2015	E	20.0	M	IRI	ASC	17	—	—	—	25.9 (7.5)	T-CE: 3.1 (0.6), T-EC: 3.3 (0.7), T-EA: 3.2 (0.9)	T-CE: 0.9, T-EC: 0.7, T-EA: −0.8
					TD	24	—	—	—	15.3 (5.9)	T-CE: 3.7 (0.7), T-EC: 3.8 (0.5), T-EA: 2.6 (0.7)	
Althaus et al., 2015	W	22.6	M	EQ and IRI	ASC	31	—	PIQ: — VIQ: — FSIQ: 105.0 (17.5)	—	24.4 (7.4)	T-CE: 8.4 (5.4), T-CE: 15.3 (5.6), T-EC: 15.8 (5.4), T-EC: 9.3 (4.9), T-EA: 12.3 (5.5	T-CE: 0.8, T-CE: 0.3, T-EC: −0.2, T-EC: 0.6, T-EA: −1.0
					TD	30	—	PIQ: — VIQ: — FSIQ: 104.4 (10.3)	—	13.0 (6.4)	T-CE: 12.7 (4.5), T-CE: 16.6 (4.9), T-EC: 14.8 (4.3), T-EC: 12.1 (4.6), T-EA: 7.6 (3.4)	
Baron-Cohen et al., 2001	W	21.9	M	RMEC	ASC	15	—	PIQ: — VIQ: — FSIQ: 115.0 (16.1)	—	34.4 (6.0)	S-CE: 21.9 (6.6)	S-CE: 1.5
					TD	103	—	PIQ: — VIQ: — FSIQ: 116.0 (6.4)	—	18.4 (6.3)	S-CE: 28.0 (3.5)	
Baron-Cohen et al., 2015	W	39.2	M	RMEC	ASC	178	—	—	—	35.7 (10.0)	S-CE: 23.5 (6.6), S-CE: 23.5 (7.1)	S-CE: 0.3, S-CE: 0.7
					TD	152	—	—	—	16.3 (6.3)	S-CE: 25.5 (4.6), S-CE: 27.4 (3.4)	
Bellebaum et al., 2014	W	28.4	M	IRI and PS	ASC	10	—	PIQ: — VIQ: — FSIQ: 110.8 (15.3)	—	41.5 (3.6)	T-CE: 23.2 (5.1), T-EC: 25.7 (5.2), S-EA: 1.0 (0.9	T-CE: 1.3, T-EC: −0.4, S-EA: 0.6
					TD	12	—	PIQ: — VIQ: — FSIQ: 111.7 (13.7)	—	13.3 (5.4)	T-CE: 29.4 (4.2), T-EC: 24.0 (3.0), S-EA: 1.7 (1.2)	
Bellesi et al., 2016	W	21.8	M	SST	ASC	19	—	PIQ: — VIQ: — FSIQ: 115.4 (8.9)	—	—	S-EA: 41.5 (15.3)	S-EA: 0.3
					TD	19	—	PIQ: — VIQ: — FSIQ: 111.0 (9.8)	—	—	S-EA: 45.7 (8.8)	
Bird et al., 2010	W	34.8	M	PS	ASC	18	—	PIQ:110.2 (16.6) VIQ:117.3 (13.4) FSIQ: 115.8 (14.6)	8.6 (3.9)	—	S-EA: −0.8 (2.7), S-EA: −5.6 (2.3), S-EA: −1.2 (3.1), S-EA: −4.6 (3.4)	S-EA: 0.9, S-EA: −0.1, S-EA: 1.0, S-EA: −0.3
					TD	18	—	PIQ:111.9 (11.8) VIQ:118.9 (7.9) FSIQ: 118.8 (11.7)	—	—	S-EA: 2.0 (3.6), S-EA: −5.9 (2.7), S-EA: 2.1 (3.4), S-EA: −5.5 (2.5)	
Bos and Stokes, 2018	E	13.2	M	IRI	ASC	24	—	—	—	28.0 (7.8)	T-CE: 13.8 (5.6), T-EC: 14.6 (5.1)	T-CE: 0.8, T-EC: 0.9
					TD	24	—	—	—	14.5 (6.1)	T-CE: 18.0 (4.1), T-EC: 18.8 (4.3)	
Brewer et al., 2015	W	33.0	M	ES	ASC	25	—	PIQ: — VIQ: — FSIQ: 107.2 (16.9)	—	26.6 (11.7)	S-EA: 1.8 (0.5)	S-EA: 0.2
					TD	22	—	PIQ: — VIQ: — FSIQ: 106.9 (16.2)	—	18.9 (8.6)	S-EA: 1.9 (0.6)	
Campbell et al., 2015	W	1.9	M	EVR	ASC	12	—	—	5.3 (1.3)	—	S-EC: 1.2 (1.2), S-EC: 0.3 (0.5), S-EA: 1.0 (1.2), S-EA: 0.3 (0.5)	S-EC: 0.7, S-EC: 1.0, S-EA: 0.9, S-EA: 1.0
					TD	57	—	—	1.6 (1.0)	—	S-EC: 1.8 (1.0), S-EC: 1.2 (0.9), S-EA: 1.8 (0.8), S-EA: 1.1 (0.9)	
Chung et al., 2016	E	17.8	M	IRI	ASC	17	—	PIQ: — VIQ: — FSIQ: 99.9 (13.4)	—	29.6 (7.3)	T-CE: 15.1 (3.9), T-EC: 14.1 (4.7), T-EA: 14.5 (4.1)	T-CE: 0.9, T-EC: 0.7, T-EA: −0.2
					TD	22	—	PIQ: — VIQ: — FSIQ: 102.6 (14.3)	—	17.5 (6.1)	T-CE: 18.4 (3.2), T-EC: 17.3 (4.1), T-EA: 13.9 (4.5)	
De Coster et al., 2017	W	31.1	F	IRI	ASC	20	—	PIQ: — VIQ: — FSIQ: 128.6 (20.9)	—	33.7 (7.7)	T-CE: 35.7 (12.0), T-EC: 41.2 (12.2), T-EA: 59.9 (7.7)	T-CE: 1.2, T-EC: 0.3, T-EA: −1.6
					TD	20	—	PIQ: — VIQ: — FSIQ: 124.5 (15.7)	—	13.9 (6.3)	T-CE: 49.0 (9.4), T-EC: 44.3 (9.5), T-EA: 46.9 (8.3)	
Deschamps et al., 2014	W	7.0	M	GEM and ST	ASC	22	—	PIQ: — VIQ: — FSIQ: 114.0 (24.8)	—	—	T-CE: −0.9 (7.7), T-CE: −0.8 (7.6), T-EC: −2.3 (13.8), T-EC: 3.3 (11.9), S-CE: 1.6 (0.7), S-EA: 2.6 (2.6), S-EA: 2.8 (2.4), S-EA: 2.0 (2.4), S-EA: 1.5 (2.1)	T-CE: 1.5, T-CE: 1.3, T-EC: 0.3, T-EC: 0.6, S-CE: 0.4, S-EA: −0.1, S-EA: −0.0, S-EA: 0.3, S-EA: 0.1
					TD	29	—	PIQ: — VIQ: — FSIQ: 119.0 (27.8)	—	—	T-CE: 9.9 (6.3), T-CE: 7.0 (4.2), T-EC: 1.3 (8.0), T-EC: 2.0 (5.3), S-CE: 1.9 (0.4), S-EA: 2.2 (2.5), S-EA: 2.8 (2.0), S-EA: 2.6 (2.3), S-EA: 1.7 (2.1)	
Dziobek et al., 2008	W	45.6	M	MET and IRI	ASC	17	16.5 (1.8)	PIQ: — VIQ: — FSIQ: 110.0 (9.0)	—	—	T-CE: 11.1 (5.7), T-EC: 16.0 (5.7), T-EA: 17.4 (6.9), S-CE: 11.9 (2.3), S-EC: 6.3 (1.6), S-EA: 6.7 (1.2)	T-CE:1.5, T-EC: 0.7, T-EA: −1.5, S-CE: 0.7, S-EC: −0.1, S-EA: −0.5
					TD	18	16.3 (1.3)	PIQ: — VIQ: — FSIQ: 112.0 (9.0)	—	—	T-CE: 18.9 (4.4), T-EC: 19.7 (5.1), T-EA: 9.1 (3.0), S-CE: 13.1 (0.9), S-EC: 6.2 (1.3), S-EA: 6.1 (1.2)	
Eyuboglu et al., 2018	W	12.3	F	ERT	ASC	41	—	—	—	—	S-CE: 10.7 (2.6), S-CE: 11.4 (3.3)	S-CE: 1.3, S-CE: 1.8
					TD	43	—	—	—	—	S-CE: 14.0 (2.3), S-CE: 16.4 (2.0)	
Fan et al., 2014	E	19.8	M	FPS	ASC	24	—	PIQ: — VIQ: — FSIQ: 107.0 (11.2)	—	30.4 (5.3)	S-EA: 1.8 (0.8), S-EA: 2.4 (1.2)	S-EA: 2.0, S-EA: 0.3
					TD	21	—	PIQ: — VIQ: — FSIQ: 111.5 (10.3)	—	19.3 (5.3)	S-EA: 3.3 (0.7), S-EA: 2.7 (0.6)	
Frey et al., 2015	W	4.0	M	SSRS	ASC	34	—	—	—	—	T-EC: 5.7 (3.9), T-EC: 8.6 (3.6)	T-EC: 0.5, T-EC: 1.1
					TD	92	—	—	—	—	T-EC: 7.6 (4.2), T-EC: 12.5 (3.6)	
Golan and Baron-Cohen, 2006	W	28.0	M	RMEC	ASC	22	—	PIQ: 115.3 (12.3) VIQ: 109.7 (10.0) FSIQ: —	—	38.2 (7.5)	S-CE: 21.3 (9.0)	S-CE: 0.4
					TD	24	—	PIQ: 112.5 (8.9) VIQ: 115.8 (13.7) FSIQ: —	—	14.0 (5.9)	S-CE: 25.3 (9.6)	
Golan et al., 2015	W	9.8	M	CAM-C	ASC	30		PIQ: 111.0 (15.3) VIQ: 112.9 (12.9) FSIQ: 113.5 (11.8)	—	19.7 (4.3)	S-CE: 15.0 (3.9), S-CE: 16.4 (3.6)	S-CE: 1.1, S-CE: 1.0
					TD	25		PIQ: 112.0 (13.3) VIQ: 114.0 (12.3) FSIQ: 114.8 (11.9)	—	3.4 (1.7)	S-CE: 19.2 (3.7), S-CE: 20.1 (3.5)	
Groen et al., 2015	W	32.4	M	EQ	ASC	42	—	PIQ: — VIQ: — FSIQ: >80	—	—	T-CE: 7.5 (5.0), T-EC: 8.4 (4.4)	T-CE: 1.2, T-EC: 1.2
					TD	685	—	PIQ: — VIQ: — FSIQ: —	—	—	T-CE: 13.2 (4.9), T-EC: 14.1 (4.9)	
Gu et al., 2015	W	26.5	M	PP	ASC	17	—	PIQ: — VIQ: — FSIQ: 109.5 (18.0)	12.0 (—)	—	S-CE: 0.9 (0.1), S-CE: 0.9 (0.1)	S-CE: 0.9, S-CE: 0.5
					TD	17	—	PIQ: — VIQ: — FSIQ: 113.5 (11.9)	—	—	S-CE: 0.9 (0.0), S-CE: 0.9 (0.1)	
Hirvelä and Helkama, 2011	W	35.6	F	IRI	ASC	41	14.0 (2.4)	—	—	—	T-CE: 13.6 (5.6), T-EC: 15.6 (5.7), T-EA: 12.8 (6.4)	T-CE: 0.7, T-EC: 0.7, T-EA: −0.6
					TD	139	13.6 (2.9)	—	—	—	T-CE: 17.0 (4.3), T-EC: 18.9 (4.1), T-EA: 10.0 (4.4)	
Klapwijk et al., 2016	W	17.1	M	EET	ASC	23	—	PIQ: — VIQ: — FSIQ: 107.1 (10.4)	—	66.7 (21.6)	S-CE: 36.2 (8.0)	S-CE: 0.5
					TD	33	—	PIQ: — VIQ: — FSIQ: 97.2 (9.2)	—	34.5 (13.9)	S-CE: 36.5 (6.0)	
Krach et al., 2015	W	22.9	M	PP	ASC	16	—	FSIQ: 117.5 (14.4) PIQ: VIQ:	6.0 (4.7)	30.1 (8.8)	S-CE: 1.7 (1.0), S-CE: 2.3 (1.1)	S-CE: −0.1, S-CE: 0.1
					TD	16	—	FSIQ: 113.3 (10.7) PIQ: VIQ:	—	11.9 (5.7)	S-CE: 1.6 (1.1), S-CE: 2.4 (1.1)	
Mathersul et al., 2013	E	39.2	M	IRI and EQ	ASC	40	15.1 (2.4)	PIQ: — VIQ: — FSIQ: 113.6 (15.6)	—	32.5 (7.5)	T-CE: 12.6 (4.9), T-CE: 4.1 (4.0), T-EC: 17.2 (6.7), T-EC: 6.6 (4.4)	T-CE: 1.2, T-CE: 2.1, T-EC: 0.5, T-EC: 0.9
					TD	33	16.1 (2.2)	PIQ: — VIQ: — FSIQ: 114.5 (21.9)	—	15.4 (5.8)	T-CE: 18.8 (5.3), T-CE: 12.9 (4.4), T-EC: 20.2 (4.1), T-EC: 10.5 (4.6)	
McDonald and Messinger, 2012	W	2.3	M	EReT	ASC	13	4.8 (1.5)	—	5.2 (2.2)	—	S-EC: 1.3 (0.5), S-EC: 1.8 (0.8), S-EC: 1.8 (1.0), S-EC: 3.0 (1.4)	S-EC: 0.4, S-EC: 0.2, S-EC: 0.6, S-EC: 0.5
					TD	25	4.9 (1.4)	—	2.2 (1.3)	—	S-EC: 1.5 (0.7), S-EC: 2.0 (0.9), S-EC: 2.7 (1.8), S-EC: 3.8 (1.8)	
McDonald et al., 2016	W	2.0	M	EReT	ASC	51	—	—	—	—	S-EC: −0.2 (1.0)	S-EC: 0.4
					TD	33	—	—	—	—	S-EC: 0.2 (0.7)	
Minio-Paluello et al., 2008	W	26.5	M	IRI	ASC	16	—	PIQ: 119.5 (13.1) VIQ: 118.7 (9.7) FSIQ: 118.9 (15.6)	—	37.0 (5.0)	T-CE: 14.0 (4.0), T-EC: 15.0 (5.0), T-EA: 12.0 (5.0)	T-CE: 0.8, T-EC: 0.9, T-EA: 0.2
					TD	20	—	PIQ: 119.9 (10.1) VIQ: 121.3 (8.3) FSIQ: 122.9 (6.9)	—	18.0 (6.0)	T-CE: 17.0 (3.0), T-EC: 19.0 (4.0), T-EA: 13.0 (5.0)	
Mul et al., 2018	W	25.7	M	QCAE and MET	ASC	26	—	PIQ: — VIQ: — FSIQ: 113.8 (12)	—	31.1 (9.3)	T-CE: 43.1 (12.6), T-EA: 29.5 (5.8), S-CE: 0.7 (0.0), S-EA: 4.6 (1.7)	T-CE: 1.5, T-EA: 0.5, S-CE: 0.0, S-EA: 0.2
					TD	26	—	PIQ: — VIQ: — FSIQ: 110.9 (13.5)	—	16.7 (6.4)	T-CE: 58.0 (5.8), T-EA: 32.1 (5.0), S-CE: 0.7 (0.1), S-EA: 5.0 (1.8)	
Murray et al., 2017	W	30.6	M	IRI and RMEC	ASC	20	—	PIQ: — VIQ: 105.1 (17.0) FSIQ: —	—	34.2 (7.4)	T-CE: 13.2 (6.1), T-EC: 17.4 (4.1), T-EA: 14.4 (5.7), S-CE: 25.0 (4.1), S-CE: 23.8 (2.4)	T-CE: 0.8, T-EC: 0.1, T-EA: −0.8, S-CE: 0.6, S-CE: 0.5
					TD	20	—	PIQ: — VIQ: 111.3 (11.5) FSIQ: —	—	15.6 (7.2)	T-CE: 17.8 (4.8), T-EC: 17.8 (2.6), T-EA: 10.3 (4.3), S-CE: 27.7 (4.3), S-CE: 24.8 (1.9)	
Newbigin et al., 2016	W	9.2	M	EReT	ASC	21	—	PIQ: 98.6 (15.1) VIQ: 95.9 (14.9) FSIQ: 96.9 (14.3)	12.3 (3.1)	—	S-EC: 1.0 (0.5), S-EC: 0.2 (0.4), S-EC: 1.4 (0.7), S-EC: 0.6 (0.6)	S-EC: 0.4, S-EC: 0.6, S-EC: −0.3, S-EC: 0.3
					TD	17	—	PIQ: 111.5 (18.9) VIQ: 114.4 (16.3) FSIQ: 114.5 (18.6)	—	—	S-EC: 1.1 (0.3), S-EC: 0.5 (0.5), S-EC: 1.2 (0.5), S-EC: 0.8 (0.7)	
Oberman et al., 2009	W	10.2	M	IRI and BEES	ASC	13	—	PIQ: — VIQ: — FSIQ: 102.8 (15.8)	13.6 (5.3)	—	T-EC: 10.9 (7.1), S-CE: 72.6 (13.8)	T-EC: 1.7, S-CE: 0.6
					TD	13	—	PIQ: — VIQ: — FSIQ: 112.5 (17.3)	—	—	T-EC: 20.6 (3.7), S-CE: 80.2 (10.6)	
Paulus et al., 2013	W	20.8	M	ES	ASC	32	—	PIQ: — VIQ: 114.5 (15.5) FSIQ: —	11.62 (3.99)	16.2 (6.5)	S-CE: 3.5 (1.5), S-EC: 3.8 (1.9), S-EC: 2.7 (1.1), S-EC: 1.1 (0.2), S-EA: 4.2 (1.7)	S-CE: 0.5, S-EC: 0.3, S-EC: 1.2, S-EC: −0.3, S-EA: 0.2
					TD	32	—	PIQ: — VIQ: 115.4 (12.4) FSIQ: —	—	7.5 (4.3)	S-CE: 4.2 (1.0), S-EC: 4.3 (1.0), S-EC: 4.0 (1.1), S-EC: 1.0 (0.1), S-EA: 4.4 (1.3)	
Peterson, 2014	W	6.4	M	EBGM	ASC	37	—	—	—	—	S-EC: 1.4 (1.1)	S-EC: 1.4
					TD	39	—	—	—	—	S-EC: 2.7 (0.7)	
Peterson et al., 2015	E	9.3	M	B-ET and EH and RMEC	ASC	34	—	PIQ: — VIQ: 93.2 (21.8) FSIQ: —	—	—	S-CE: 5.8 (2.1), S-CE: 9.5 (1.7), S-CE: 5.2 (1.7), S-CE: 9.8 (1.6), S-EC: 9.2 (2.5)	S-CE: 0.6, S-CE: 0.2, S-CE: 1.0, S-CE: 0.3, S-EC: 1.8
					TD	41	—	PIQ: — VIQ: 103.6 (18.7) FSIQ: —	—	—	S-CE: 7.0 (1.7), S-CE: 9.9 (1.2), S-CE: 7.2 (2.2), S-CE: 10.3 (1.2), S-EC: 13.4 (2.2)	
Ponnet et al., 2004	W	21.5	M	RMEC	ASC	19	—	PIQ: 104.1 (18.1) VIQ: 108.3 (14.0) FSIQ: 106.6 (15.1)	—	—	S-CE: 35.3 (18.7)	S-CE: 0.5
					TD	19	—	PIQ: 110.6 (14.0) VIQ: 116.0 (18.4) FSIQ: 114.1 (15.8)	—	—	S-CE: 45.0 (19.5)	
Poustka et al., 2010	W	13.6	M	MET	ASC	15	—	PIQ: 97.0 (10.3) VIQ: 107.0 (12.7) FSIQ: —	—	—	S-EC: 4.9 (1.4), S-EA: 4.3 (1.6)	S-EC: −0.3, S-EA: −0.2
					TD	15	—	PIQ: 111.0 (10.0) VIQ: 110.0 (14.5) FSIQ: —	—	—	S-EC: 4.5 (1.3), S-EA: 4.0 (1.4)	
Pouw et al., 2013	W	11.6	M	EmQ	ASC	67	—	PIQ: — VIQ: — FSIQ: >80	—	—	T-CE: 2.2 (0.5), T-EC: 1.6 (0.4), T-EA: 1.6 (0.5)	T-CE: 0.7, T-EC: 0.3, T-EA: 0.0
					TD	66	—	PIQ: — VIQ: — FSIQ: >80	—	—	T-CE: 2.5 (0.4), T-EC: 1.7 (0.5), T-EA: 1.6 (0.5)	
Rogers et al., 2007	W	42.4	M	IRI	ASC	21	16.7 (1.6)	PIQ: — VIQ: — FSIQ: 121.8 (6.8)	—	—	T-CE: 10.5 (6.4), T-EC: 16.9 (6.5), T-EA: 15.8 (8.0)	T-CE: 1.5, T-EC: 0.5, T-EA: −0.9
					TD	21	16.1 (1.7)	PIQ: — VIQ: — FSIQ: 120.6 (9.0)	—	—	T-CE: 18.9 (4.3), T-EC: 20.0 (4.7), T-EA: 9.6 (5.2)	
Scheeren et al., 2013	W	11.2	M	SOER	ASC	151	—	PIQ: — VIQ: 105.3 (13.0) FSIQ: —	5.7 (4.1)	—	S-EC: 0.2 (0.2), S-EC: 0.5 (0.3)	S-EC: −0.2, S-EC: 0.8
					TD	50	—	PIQ: — VIQ: 107.2 (12.2) FSIQ: —	—	—	S-EC: 0.1 (0.2), S-EC: 0.8 (0.2)	
Schneider et al., 2013	W	31.4	M	ES	ASC	28	12.7 (0.8)	PIQ: — VIQ: — FSIQ: 109.1 (9.1)	—	37.7	S-CE: 58.6 (31.1), S-CE: 94.8 (6.5), S-CE: 0.8 (0.5), S-EA: 0.8 (0.4), S-EA: 1.0 (0.4)	S-CE: 0.7, S-CE: 0.3, S-CE: −0.1, S-EA: 0.6, S-EA: −2.6
					TD	28	12.9 (0.3)	PIQ: — VIQ: — FSIQ: 114.1 (9.6)	—	10.1	S-CE: 78.2 (21.0), S-CE: 96.5 (5.1), S-CE: 0.8 (0.6), S-EA: 1.0 (0.4), S-EA: 0.2 (0.2)	
Schwenck et al., 2012	W	12.2	M	AST and MT and VST	ASC	55	—	PIQ: — VIQ: — FSIQ: 102.6 (15.5)	—	—	S-CE: 0.4 (0.2), S-CE: 6.3 (2.6), S-EC: 0.5 (0.3)	S-CE: 0.5, S-CE: −0.0, S-EC: −0.3
					TD	67	—	PIQ: — VIQ: — FSIQ: 105.7 (14.5)	—	—	S-CE: 0.5 (0.2), S-CE: 6.2 (2.5), S-EC: 0.4 (0.2)	
Senland and Higgins-D'Alessandro, 2013	W	15.3	M	IRI	ASC	16	—	—	—	32.4 (6.3)	T-CE: 14.9 (4.5), T-EC: 20.6 (3.8), T-EA: 11.6 (6.2)	T-CE: 0.4, T-EC: 0.0, T-EA: −0.9
					TD	16	—	—	—	12.6 (4.9)	T-CE: 16.9 (5.6), T-EC: 20.6 (4.4), T-EA: 7.0 (3.2)	
Senland and Higgins-D'Alessandro, 2016	W	19.3	M	IRI	ASC	22	—	PIQ: — VIQ: — FSIQ: 104.4 (15.2)	—	—	T-CE: 15.5 (5.7), T-EC: 19.1 (6.3), T-EA: 11.9 (4.8)	T-CE: 0.8, T-EC: 0.1, T-EA: −0.7
					TD	22	—	PIQ: — VIQ: — FSIQ: 110.9 (8.8)	—	—	T-CE: 19.5 (4.5), T-EC: 19.6 (4.7), T-EA: 8.7 (4.0)	
Silani et al., 2008	W	35.1	M	IRI	ASC	14	—	PIQ: — VIQ: — FSIQ: 117.6 (13.5)	9.8 (2.4)	—	T-CE: 9.8 (3.1), T-EC: 16.4 (4.2), T-EA: 14.6 (6.3)	T-CE: 1.6, T-EC: 0.3, T-EA: −0.6
					TD	15	—	PIQ: — VIQ: — FSIQ: 119.6 (11.4)	—	—	T-CE: 16.1 (4.5), T-EC: 17.7 (4.2), T-EA: 11.3 (5.0)	
Sucksmith et al., 2013	W	35.1	F	KDEF	ASC	329	—	PIQ: 52.5 (4.1) VIQ: — FSIQ: —	—	—	S-CE: 16.6 (1.1)	S-CE: 0.9
					TD	187	—	PIQ: 52.7 (3.6) VIQ: — FSIQ: —	—	—	S-CE: 17.6 (1.2)	
Thaler et al., 2018	W	25.0	M	IRI	ASC	16	—	PIQ: — VIQ: — FSIQ: 103.5 (12.7)	—	—	T-CE: 24.0 (4.4), T-EC: 21.8 (3.5), T-EA: 19.6 (5.0)	T-CE: 0.5, T-EC: 0.4, T-EA: −0.2
					TD	16	—	PIQ: VIQ: FSIQ: 111.8 (11.1)	—	—	T-CE: 26.2 (3.4), T-EC: 23.1 (2.8), T-EA: 18.5 (4.5)	
Vyas et al., 2017	W	20.9	F	IRI	ASC	20	—	—	—	31.4 (3.7)	T-CE: 15.4 (4.3), T-EC: 17.6 (5.9), T-EA: 13.1 (4.2)	T-CE: 0.9, T-EC: 0.3, T-EA: −0.7
					TD	60	—	—	—	7.2 (2.1)	T-CE: 20.0 (5.4), T-EC: 19.6 (6.1), T-EA: 10.1 (3.9)	
Zalla et al., 2011	W	27.2	M	FpRT	ASC	20	14.1 (3.4)	PIQ: 91.4 (20.5) VIQ: 100.3 (19.3) FSIQ: 96.0 (20.9)	—	—	S-CE: 4.2 (2.1)	S-CE: 1.4
					TD	33	13.6 (3.0)	PIQ: 99.5 (12.1) VIQ: 100.8 (11.7) FSIQ: 102.0 (13.2)	—	—	S-CE: 7.4 (2.4)	
Zuluaga Valencia et al., 2018	W	8.0	M	ERT	ASC	10	—	—	—	—	S-CE: 13.1 (2.0)	S-CE: 0.8
					TD	10	—	—	—	—	S-CE: 14.6 (1.8)	
Yu et al., 2017	E	20.0	F	IRI	ASC	274	—	—	—	>24	T-EC: 17.0 (3.8), T-EA: 9.0 (4.2)	T-EC: 0.3, T-EA: −0.8
					TD	300	—	—	—	<17	T-EC: 18.1 (3.3), T-EA: 6.0 (3.9)	

*Only the first author and year are listed. E, Eastern culture; W, Western culture; Age, Average age of subjects in ASC group and TD group; M, Masculine; F, Feminine; IRI, Interpersonal Reactivity Index; EQ, Empathy Quotient; RMEC, Reading the Mind in the Eyes; MET, Multifaceted Empathy Test; SST, Social Strategy Task; ERT, Emotion Recognition Test; KDEF, Karolinska Directed Emotional Faces task; PS, Pain stimulation; ES, Emotive statements; EVR, Empathic video records; GEM, Griffith Empathy Measure; ST, Story task; FPS, Facial pain scale; SSRS, Social Skills Rating System; CAM, Cambridge Mindreading Face-Voice Battery; CAM-C, The Cambridge Mindreading Face-Voice Battery for Children; PP, Painful photographs; EET, Explicit empathy task; EReT, Empathic responding task; FpRT, Faux Pas Recognition Test; EmQ, Empathy questionnaire; QCAE, Questionnaire of Cognitive and Affective Empathy; BEES, Basic Emotional Empathy Scale; EBGM, Empathic behavior global measure; B-ET, Body-emotion test; EH, Empathic helpfulness; SOER, Structured observation of empathic responsiveness; ES, Emotional stories; AST, Animated shapes task; MT, Morphing task; VST, Video sequences task; ADOS, Autism Diagnostic Observation Schedule; AQ, Autism Spectrum Quotient Questionnaire; —, Not reported; FSIQ, Full scale intelligence quotient; PIQ, Performance intelligence quotient; VIQ, Verbal intelligence quotient; Values given as M (SD); T-CE, Trait-cognitive empathy; T-EC, Trait-empathic concern; T-EA, Trait-empathic accuracy; S-CE, State-cognitive empathy; S-EC, State-empathic concern; S-EA, State-empathic accuracy; ASC, Autism spectrum conditions group; TD, Typically developing group, namely, the controls or normal individuals. If the same outcome variables are shown several times in one study, it means that the study used repeated measures or adopted completely different tasks for one outcome variable*.

As shown in Figure 1, a total of 12,349 studies in Chinese and foreign languages were initially retrieved (as of November 2018), and 51 studies were included in the meta-analysis according to the criteria above. Among them, 44 studies were Western studies, and seven were Eastern studies; 45 studies were predominantly male, and six studies were predominantly female. These studies involved 2,095 individuals with ASCs and 2,869 controls without ASCs who came from 17 countries and regions including Australia, the United States, the United Kingdom, Germany, China, etc. Their average age was 21.15 years (*SD* = 11.39) with an overall range of 1.90–48.60 years [ASC: 20.92 (*SD* = 11.19), range of 1.90–42.90; TD: 21.36 (*SD* = 11.67), range of 1.90–48.60]. Moreover, according to the encoding results of the outcome variables, there were 21 studies with T-CE (containing 24 independent effects), 23 studies with T-EC (including 28 independent effects), 17 studies with T-EA (consisting of 17 independent effects), 21 studies with S-CE (comprising 33 independent effects), 11 studies with S-EC (containing 21 independent effects), and 12 studies with S-EA (including 21 independent effects).

### Meta-Analysis Process

#### Effect Quantity Calculation

The correction quantity of Cohen's *d*, namely, Hedge's *g* (or the standardized mean difference) (Olde Dubbelink and Geurts, 2017), was adopted as the index of empathy impairment of ASC individuals. It was directly obtained by CMA V2.2.064 software [Biostat; July 27, 2011; USA +1 (201) 541–5688] after the input of the sample size, mean value, and standard deviation. In our study, a positive *g*-value indicates that the empathy ability or reaction force is higher in the TD group than in the ASC group, that is, the empathy of the ASC group is impaired; conversely, a negative *g*-value indicates that the empathy of the ASC group is equal to or better than the empathy of the TD group.

#### Model Selection and Heterogeneity Testing

According to a previous literature review, both the empathy and symptom diagnosis of ASCs are affected by the cultural background, gender, and age of the subjects to some extent. This means that the measurement results for empathy impairment in ASC individuals not only contain the true score of empathy impairment but also may include variance caused by other variables. Therefore, a random-effects model was selected for the meta-analysis (Borenstein et al., 2010).

Meanwhile, the results of the *Q, I*^2^, and *H*-tests were used to confirm the rationality of the selected random-effects model. The *Q*-value was subject to the χ^2^ distribution with a *df* of k-1 (with k representing the number of studies included in the meta-analysis); a larger *Q*-value results in a smaller corresponding *p*-value, and *Q* indicates heterogeneity if *p* < α (usually 0.05). The *I*^2^-test indicates the proportion of true variance in the total variance. The formula is *I*^2^ = 100% × *(Q–df)*/*Q*, and the critical points of low, medium and high heterogeneity are 25.0, 50.0, and 75.0%, respectively. Generally, there is significant heterogeneity when *I*^2^ > 50% (Higgins et al., 2003). *H* is the correction value for the *Q*-value with the formula *H* = *[Q/(K*−*1)]*^−1^; a value >1.5 indicates a high degree of heterogeneity.

#### Publication Bias

The publication bias test was conducted to evaluate whether the published literature could systematically and comprehensively represent all literature completed in a specific field (Rothstein et al., 2006). To confirm that the literature in this meta-analysis was comprehensively and systematically included, Rosenthal's classic Failsafe-*N* method and Egger's regression intercept method were used for testing, and the funnel plot was also supplemented for intuitive explanation.

## Results

### Publication Bias Test

**Figure 2:**
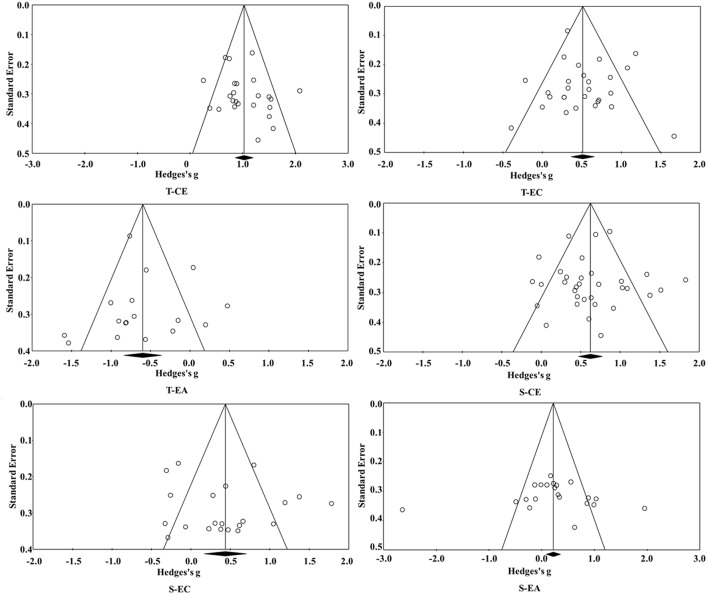
Funnel plots of the publication bias of the empathy components.

According to the funnel plots in Figure 2, the Hedge's *g* of the empathy components was distributed symmetrically on both sides of the total *g*-value, which indicates that there was basically no publication bias. The results of Rosenthal's classic Failsafe-*N* and Egger's regression intercept (Table 2) also showed that there was no publication bias in T-CE, T-EC, T-EA, S-CE, or S-EC (i.e., the FSR values were all >1, and the *p*-values were all >0.05). Although the results of Rosenthal's classic Failsafe-*N* test for S-EA showed that there was a publication bias (*FSR* < 1), the result of Egger's regression intercept was negative (*p* > 0.05), which demonstrates that there was no serious publication bias for S-EA.

**Table 2:** Publication bias test.

**Outcome variables**	***K***	**Classic failsafe-*N***	***FSR***	**Egger's intercept**	***SE***	**95% CI**	***p***
T-CE	24	1874	14.415	1.326	1.108	[−0.971, 3.623]	0.244
T-EC	28	801	5.340	0.262	0.729	[−1.236, 1.761]	0.722
T-EA	17	396	4.168	0.170	1.030	[−2.025, 2.365]	0.871
S-CE	33	1819	10.394	−0.055	0.772	[−1.629, 1.520]	0.944
S-EC	21	253	2.200	1.458	1.933	[−2.589, 5.505]	0.460
S-EA	21	37	0.322	0.099	4.419	[−9.150, 9.347]	0.982

*k represents the number of independent effects; FSR, failsafe ratio, whose formula is FSR = N_fs_/(5k + 10); 95% CI refers to the 95% confidence interval of Egger's intercept*.

### Heterogeneity Test

As seen in Table 3, the *Q*-values of all components of empathy were significant (*p* < 0.05), the *I*^2^-values were >50%, and the *H*-values were between 1.5 and 2.4, which indicate that the components of empathy had high heterogeneity and that the selected random-effects model was reasonable.

**Table 3:** Heterogeneity test.

**Outcome variables**	***N***	***df***	***Q***	***I*^**2**^**	**Tau-squared**	***H***
T-CE	1837	23	50.100^***^	54.092	0.092	1.5
T-EC	2511	27	66.917^***^	59.652	0.083	1.6
T-EA	1490	16	60.633^***^	73.612	0.163	1.9
S-CE	2347	32	106.447^***^	69.938	0.118	1.8
S-EC	832	20	104.112^***^	80.790	0.291	2.3
S-EA	585	20	119.330^***^	83.240	0.477	2.4

N is the sum of the sample size of the ASC and TD groups;

*** *p < 0.001*.

### Analysis of the Impairment and Sensitivity of Empathy Among ASC Individuals

**FIGURE 3:**
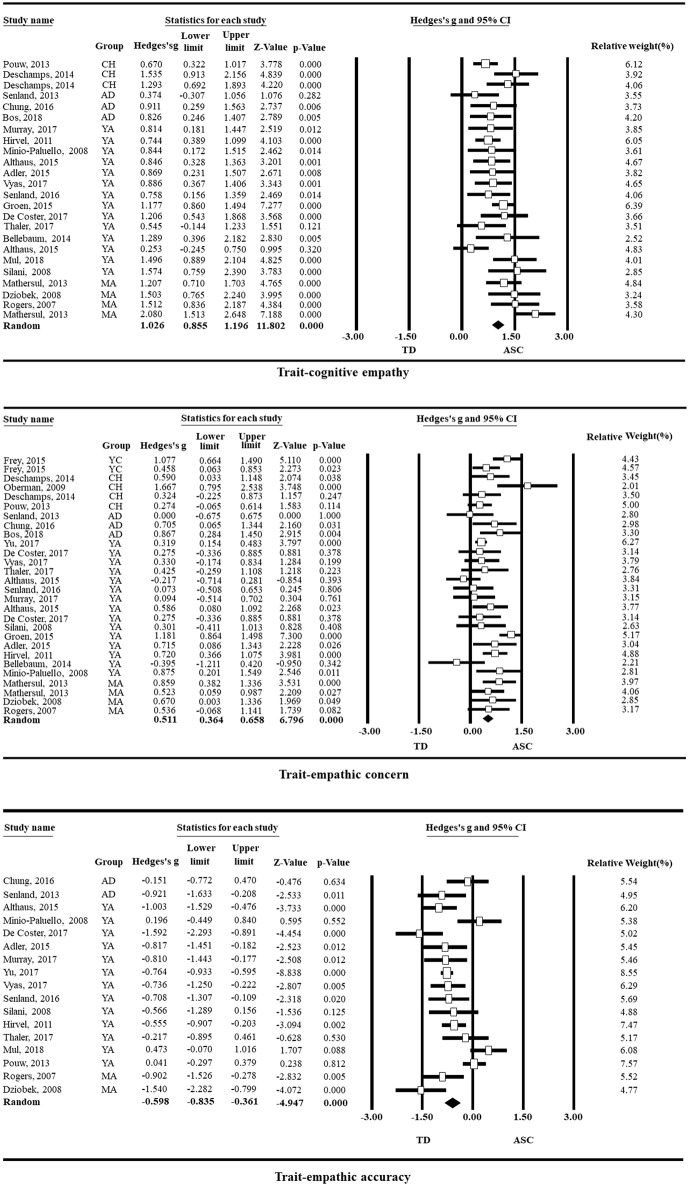
Forest plots of the impairment of the empathy components among ASC individuals (TO, Toddlers, aged 0–3 years; YC, Young children, aged 4–6 years; CH, Children, aged 7–12 years; AD, Adolescence, aged 13–18 years; YA, Young adults, aged 19–39 years; MA, Mature adults, aged of 40–59 years; SE, Seniors, aged ≥ 60 years).

The empathy impairment of ASC individuals was analyzed by taking the standardized mean difference *g* as the effect quantity, and the results are shown in Figure 3.

Generally, it is believed that there is no difference between the experimental and the control groups when the 95% confidence interval horizontal line of Hedge's *g* intersects the invalid line (the vertical line with the horizontal coordinate of 0) on the forest map (Higgins and Green, 2011). In our study, as indicated by the forest maps (detailed in Figure 3), the degree of the confidence intervals' horizontal lines for T-CE, T-EC, S-CE, and S-EC that do not intersect the invalid line accounts for relatively large proportions of the lines, which are distributed on the right side of the invalid line in accordance with the overall effect (black diamond). This result indicates that the components of T-CE, T-EC, S-CE, and S-EC are impaired in ASC individuals from an intuitively visual perspective; i.e., the scores of T-CE, T-EC, S-CE, and S-EC were significantly lower among ASC individuals than among typically developing individuals. In terms of T-EA, the trait empathic accuracy ability of ASC individuals was visually better than the trait empathic accuracy of typically developing individuals, although most of the confidence interval horizontal lines do not intersect with the invalid line, because most of these confidence interval horizontal lines were distributed to the left side of the invalid line in accordance with the overall effect. The S-EA of ASC individuals is intact because the confidence interval lines of S-EA that do not intersect with the invalid line are evenly distributed on both sides of the invalid line, and the overall diamond effect intersects with the invalid line. The results above are further supported by the results of Hedge's *g*-test (the total effect). Specifically, Hedge's *g*-values for T-CE, T-EC, T-EA, S-CE, S-EC, and S-EA are 1.026 (*p* < 0.001), 0.511 (*p* < 0.001), −0.598 (*p* < 0.001), 0.622 (*p* < 0.001), 0.430 (*p* < 0.01), and 0.222 (*p* > 0.05), respectively, which show that the T-CE, T-EC, S-CE, and S-EC for ASC individuals are impaired, whereas the S-EA remains intact, and T-EA is superior to the T-EA of neurotypical individuals. In addition, overall, both trait empathy (*g* = 0.417, *p* < 0.05) and state empathy (*g* = 0.456, *p* < 0.05) are impaired in ASC individuals.

The sensitivity analysis of the impairment of the empathy components in ASC individuals (as detailed in Table 4) shows the following. The heterogeneity of T-CE decreased by 38.721% after the deletion of four studies, and *g* changed from 1.026 (*p* < 0.001) to 1.035 (*p* < 0.001). The heterogeneity of T-EC decreased by 38.783% after the deletion of four studies, and *g* changed from 0.511 (*p* < 0.001) to 0.492 (*p* < 0.001). The heterogeneity of T-EA decreased by 55.915% after the deletion of five studies, and *g* changed from −0.598 (*p* < 0.001) to −0.717 (*p* < 0.001). The heterogeneity of S-CE decreased by 32.127% after the deletion of six studies, and *g* changed from 0.622 (*p* < 0.001) to 0.595 (*p* < 0.001). The heterogeneity of S-EC decreased by 32.743% after the deletion of seven studies, and *g* changed from 0.430 (*p* < 0.01) to 0.407 (*p* < 0.01). Finally, the heterogeneity of S-EA decreased by 64.696% after the deletion of five studies, and *g* changed from 0.222 (*p* > 0.05) to 0.156 (*p* > 0.05). Thus, no matter how the heterogeneity changed, the deficits of the components of empathy in ASC individuals were relatively stable (Because the intervals between the high, medium, and low ranges of *I*^2^ correspond to 25%, when the change of *I*^2^ exceeds 25% and the significance of the core index of the outcome variable is still consistent with the original, it can generally be understood that the effect quantity of the outcome variable is relatively stable).

**Table 4:** Analysis of the impairment and sensitivity of empathy in ASC individuals.

**Outcome variables**	**g**	**95% CI**	**Number of deleted studies**	***I*^**2**^ before deletion**	***I*^**2**^ after deletion**	***g* after deletion**
T-CE	1.026^***^	[0.855, 1.196]	4	54.092	15.371	1.035^***^
T-EC	0.511^***^	[0.364, 0.658]	4	59.652	20.869	0.492^***^
T-EA	−0.598^***^	[−0.835, −0.361]	5	73.612	17.697	−0.717^***^
S-CE	0.622^***^	[0.472, 0.771]	6	69.938	37.811	0.595^***^
S-EC	0.430^**^	[0.168, 0.691]	7	80.790	48.047	0.407^***^
S-EA	0.222	[−0.103, 0.547]	5	83.240	18.544	0.156

*g is the total effect quantity; 95% CI is the 95% confidence interval of the effect quantity g. The deleted studies were all randomly selected*.

** *p < 0.01*,

*** *p < 0.001*.

### Moderating Effects Analysis

The univariate regression model (Ren et al., 2018) was used to investigate the moderating effects of culture, gender, and age on the deficits of the empathy components in ASC individuals. To control the insufficient accuracy of the overall effect of a certain variable caused by the small number of independent effects at a certain variable level, according to a previous study (Bar-Haim et al., 2007), we did not analyse these moderator variables with the number of independent effects at a variable level <4. The results (as detailed in Table 5) show that culture had no moderating effect on T-CE (*B* = 0.264, *p* > 0.05), T-EC (*B* = −0.082, *p* > 0.05), or S-CE (*B* = −0.004, *p* > 0.05). Gender had a significant moderating effect on T-EC (*B* = −0.201, *p* < 0.05), T-EA (*B* = −0.349, *p* < 0.01), and S-CE (*B* = 0.432, *p* < 0.001). With increasing age, the impairment of T-CE (*B* = 0.015, *p* < 0.01) and the advantage of T-EA (*B* = −0.017, *p* < 0.05) increased in ASC individuals, whereas the impairment of S-EC (*B* = −0.018, *p* < 0.01) and S-EA (*B* = −0.012, *p* < 0.05) were alleviated in ASC individuals.

**Table 5:** Meta-regression analysis of the moderating variables.

**Outcome variables**	**Regulating variables**		***k***	***g***	***B***	***SE***	**95% CI**	***z***	***p***	***Q_***M***_***	***P_***M***_***
T-CE	Culture	E	5	1.190	0.264	0.146	[−0.023, 0.550]	1.805	0.071	3.258	0.071
		W	19	0.976							
	Gender	F	3	0.857	—	—	—	—	—	—	—
		M	21	1.047							
	Age	—	24	1.026	0.015	0.005	[0.005, 0.026]	2.793	0.005	7.802	0.005
T-EC	Culture	E	6	0.584	−0.082	0.089	[−0.256, 0.092]	−0.923	0.356	0.851	0.356
		W	22	0.472							
	Gender	F	5	0.384	−0.201	0.089	[−0.375, −0.027]	−2.265	0.024	5.129	0.024
		M	23	0.538							
	Age	—	28	0.511	0.004	0.004	[−0.004, 0.012]	1.005	0.315	1.010	0.315
T-EA	Culture	E	3	−0.646	—	—	—	—	—	—	—
		W	14	−0.605							
	Gender	F	4	−0.803	−0.349	0.110	[−0.564, −0.134]	−3.183	0.001	10.130	0.001
		M	13	−0.509							
	Age	—	16	−0.598	−0.017	0.007	[−0.031, −0.004]	−2.486	0.012	6.180	0.012
S-CE	Culture	E	4	0.541	−0.004	0.112	[−0.224, 0.216]	−0.035	0.972	0.001	0.972
		W	29	0.633							
	Gender	F	4	1.110	0.432	0.079	[0.277, 0.588]	5.458	0.000	29.795	0.000
		M	29	0.527							
	Age	—	33	0.622	−0.002	0.003	[−0.008, 0.005]	−0.477	0.633	0.227	0.633
S-EC	Culture	E	0	—	—	—	—	—	—	—	—
		W	21	0.430							
	Gender	F	0	—	—	—	—	—	—	—	—
		M	21	0.430							
	Age	—	21	0.430	−0.018	0.007	[−0.031, −0.004]	−2.600	0.009	6.760	0.009
S-EA	Culture	E	2	1.121	—	—	—	—	—	—	—
		W	19	0.131							
	Gender	F	1	0.626	—	—	—	—	—	—	—
		M	20	0.204							
	Age	—	21	0.222	−0.012	0.005	[−0.023, −0.001]	−2.214	0.027	4.903	0.027

*Western culture (w) is the reference variable for culture; male (m) is the reference variable for gender; k is the number of effects; B is the slope of the regression line; SE is the standard error of B; 95% CI is the 95% confidence interval of slope B; Q_M_ is the total variance of the single factor regression model; P_M_ is the significance of Q_M_*.

## Discussion

In our study, the meta-analysis technique was used to comprehensively investigate the component specificity of the empathy impairment of ASC individuals based on a review of the relevant literature. We also examined the conditional heterogeneity of the empathy impairment in ASC individuals through a moderating effect test.

### Component Specificity of Empathy Impairment in ASC Individuals

Consistent with the results of most previous studies, we also found that T-CE (Mazza et al., 2014; Rueda et al., 2015; Senland and Higgins-D'Alessandro, 2016; De Coster et al., 2017; Murray et al., 2017; Vyas et al., 2017; Bos and Stokes, 2018; Mul et al., 2018), T-EC (Hirvelä and Helkama, 2011; Adler et al., 2015; Chung et al., 2016), S-CE (Dziobek et al., 2008), and S-EC (Campbell et al., 2015) are impaired in ASC individuals. Conversely, the T-EA is significantly better in ASC individuals than in typically developing individuals (Senland and Higgins-D'Alessandro, 2013, 2016; Adler et al., 2015; Althaus et al., 2015; De Coster et al., 2017; Murray et al., 2017; Vyas et al., 2017), and the S-EA of ASC individuals is as good as the S-EA of typically developing individuals (Poustka et al., 2010; Bellebaum et al., 2014). Of course, the conclusions of some studies are different from our study, such as the findings of intact T-CE (Althaus et al., 2015; Chung et al., 2016), S-CE (Bird et al., 2010; Schneider et al., 2013), S-EC (Poustka et al., 2010; Bellebaum et al., 2014), and T-EA (Mul et al., 2018; Thaler et al., 2018) in ASC individuals. However, given the situational dependence of empathy itself (Chen and Liu, 2016; Powell and Roberts, 2017) and the conditional heterogeneity of empathy impairment in ASC individuals, these different conclusions are understandable.

The results above not only indicate that the empathy impairment of ASC individuals is component specific but also explain a series of controversies about the empathy impairment of ASC individuals. When generally discussed, the empathic integrity of ASC individuals, whether evaluated by theoretical research (Bird and Viding, 2014; Guilé, 2014) or empirical research (Rigby et al., 2018; Tavassoli et al., 2018), is believed to be poorer than that of typically developing individuals. However, when cognitive empathy and affective empathy are analyzed separately, it is found that the trait-affective empathy of ASC individuals is intact (Mazza et al., 2014; Rueda et al., 2015; Senland and Higgins-D'Alessandro, 2016; De Coster et al., 2017; Vyas et al., 2017; Bos and Stokes, 2018; Mul et al., 2018). When further distinguishing the trait-affective empathy into T-EC and T-EA, however, it was found that ASC individuals were only impaired regarding T-EC (Hirvelä and Helkama, 2011; Adler et al., 2015; Chung et al., 2016) and that their T-EA is not only unimpaired but is actually superior to that of typically developing individuals (Adler et al., 2015; Althaus et al., 2015; Senland and Higgins-D'Alessandro, 2016; De Coster et al., 2017; Murray et al., 2017; Vyas et al., 2017). This result shows that if T-EC and T-EA are not differentiated or are combined into trait-affective empathy, the advantages of T-EA in ASC individuals could potentially compensate for the deficit of T-EC, which creates the illusion of intact trait-affective empathy. Meanwhile, because impairment of the T-CE is relatively severe in ASC individuals (the total effect *g*-value is 1.026, which is much greater than the other effect values), if the T-CE and trait-affective empathy are not discriminated, this creates the appearance of overall impaired empathy among ASC individuals. Therefore, it is necessary to distinguish among the specific components of empathy in the study of empathy impairment in ASC individuals.

The present study also indicates that the empathic deficit of ASC individuals may be isomorphic between the trait empathy level and state empathy level, although the empathic accuracy component has different manifestations at these two levels. The measurement of trait empathy is mostly based on scales with good reliability and validity. In contrast, the measurement of state empathy is more dependent on social situation stories and social stimulus pictures (Baron-Cohen et al., 2015; Cassidy et al., 2015; Krach et al., 2015; Rigby et al., 2018), especially the measurement of S-EA (Krach et al., 2015; De Coster et al., 2017), which is more dependent on the accuracy and integrity of social stimulus processing (Cuff et al., 2014). Meanwhile, the impairment of social stimulus processing is universal in ASC individuals (Happé and Ronald, 2008; Rigby et al., 2018), which may interfere with the extent to which ASC individuals share emotions with others. Therefore, ASC individuals do not show similar advantages to the advantages observed for T-EA in existing S-EA measurement tasks.

In addition, the components of empathy are not independent of one another. For example, Eerola et al. (2016) and Zickfeld et al. (2017) found that T-CE can influence individual empathic concern. Zhao et al. (2018) also found that T-CE significantly predicted not only T-EC but also S-EC together with T-EC. Moreover, studies have suggested that cognitive empathy and empathic concern together modulate empathic accuracy to some extent (Klimecki et al., 2013; Stellar et al., 2015). This means that the T-EA among ASC individuals is probably no different from the T-EA of typically developing individuals because a higher level of T-EA in ASC individuals may be caused by its unprocessed modulation due to the impaired T-CE and T-EC in ASC individuals, which coincides with the view of Zhao et al. (2018). Specifically, as higher-order empathy components, T-CE and T-EC are more similar to a type of emotion regulation strategy and could turn the aversive arousal states caused by T-EA into relatively calm states and strengthen the positive affect. However, the actual situation remains to be further explored.

### Moderating Effects of Empathy Impairment in Individuals With ASCs

#### Culture

Although cultural differences in empathy (Chentsova-Dutton and Tsai, 2010; Lin et al., 2010; Cheon et al., 2013) and autistic traits (Freeth et al., 2013; Zhao et al., 2018) have been confirmed by many studies, it is still difficult to obtain a good explanation for the cross-regional and transnational differences in the pathological symptoms of ASCs from a cultural perspective (Mandy et al., 2014). According to existing studies, the causes of cultural differences in the pathological symptoms of ASCs may be related to the different conventional standards of social behavior in different cultures (Mandy et al., 2014). Specifically, it is likely that different behavioral reference criteria for ASC identification in different cultures lead to potential cultural differences in the overall symptom score of ASCs.

However, we found that there were no significant differences between Eastern and Western cultures in the impairment of T-CE, T-EC, and S-CE in ASC individuals. One possible reason is that the potential cultural differences between the East and West in the empathy impairment of ASC individuals are masked by the cultural differences between the East and West in empathy itself and in the behavioral reference standards for ASC identification. Specifically, compared with Western individualistic cultures, Eastern collectivistic cultures emphasize the relationship between the self and others (Hua and Tan, 2012), and individuals in Eastern cultures also have stronger empathy and empathic responsiveness compared with those in Western cultures (Chentsova-Dutton and Tsai, 2010; Lin et al., 2010; Cheon et al., 2013). Compared with the standards in Western individualistic cultures, the criteria for the identification of ASCs related to empathic behavior in Eastern collectivist cultures are relatively high; therefore, as an index of the empathy impairment of ASC individuals, taking the distance between a corresponding reference standard for the empathic behavior of ASC individuals in Eastern and Western cultures vs. some universal level may lead to no obvious cultural difference. Of course, it is possible that the cultural differences in the empathy impairment of ASC individuals may have component specificity, which may be reflected in empathy components other than the T-CE, T-EC, and S-CE. Alternatively, there may be no cultural differences in empathy and autism symptom severity, with the cultural differences in empathy and autism symptoms revealed by previous studies simply being the result of bias in the measurement tools. For example, when revising the English version of the IRI in a Chinese cultural background, six inappropriate items needed to be deleted (Zhang et al., 2010). As another example, in the English version of the ADOS, the absence of eye contact and pointing behaviors is regarded as an important indicator of autism, whereas eye contact with adults and pointing with the index finger is considered to be inappropriate in the Chinese culture (Harris et al., 2013). However, whether there are cultural differences in the empathy impairment of individuals with autism, and if so, what components of empathy might be involved in them, are still worthy of further study.

#### Gender

According to previous studies, the pathological symptoms of ASCs have gender differences in both structure and degree. Structurally, females with ASCs exhibit fewer repetitive behaviors (Sipes et al., 2011; Frazier et al., 2014; Wilson et al., 2016) and more intellectual impairments than males (Fombonne, 2009). At the degree level, female ASC individuals have more attention preference for social stimuli (Chawarska et al., 2016) and less social communication impairment than males (Hartley and Sikora, 2009; Zwaigenbaum et al., 2012). However, neither gender differences in structure nor gender differences in degree are absolute; they are affected by a series of other factors, such as sample size and age (Lawson et al., 2018).

Our results showed that there are fewer T-EC deficits and more T-EA advantages in female ASC patients than in male ASC patients. This may be the manifestation of gender differences in the pathological symptoms of ASCs at the degree level (Hartley and Sikora, 2009; Zwaigenbaum et al., 2012; Chawarska et al., 2016), or it may be the embodiment of the extreme male brain dominance of autism in terms of gender (Baron-Cohen et al., 2005). That is, autism represents an extreme of the male pattern characterized by impaired empathizing and enhanced systemizing. Of course, it may also be related to the more prevalent autism traits of male individuals (Freeth et al., 2013; Zhao et al., 2018). In terms of S-CE, the greater impairment of females may be related to the greater intellectual impairment of females with ASCs (Fombonne, 2009). Because cognitive empathy is involved in individual perspective taking and mind switching (Cuff et al., 2014), the impairment of S-CE may be related to the deficit of immediate intellectual activity to some extent.

#### Age

In previous studies, both the age effect of empathy and the pathological symptoms of ASCs were controversial. Studies have suggested that empathy (Sze et al., 2012; Peterson, 2014) and the pathological symptoms of autism (Powell et al., 2017) increase with age, whereas other studies have argued the opposite (Phillips et al., 2002; Ding and Lu, 2016; Tillmann et al., 2018). Moreover, some studies have found that the age effect of empathy is component specific (Richter and Kunzmann, 2011); that is, individual empathic concern and empathic accuracy increased with age, whereas cognitive empathy decreased.

Interestingly, our study found that the empathy impairment of ASC individuals showed an age effect similar to the age effect of the development of empathy in typically developing individuals. Specifically, with increasing age, the impairment of S-EC and S-EA in ASC individuals was alleviated, whereas the impairment of T-CE was intensified. Accordingly, with the increase in age and enrichment of social experience, the affective empathy impairment of ASC individuals will be alleviated at the state level, whereas the cognitive empathy ability of ASC individuals does not show the same age trend that normal individuals should have, which results in the further widening of the differences between the two. This indicates not only that the age effect of empathy impairment in ASC individuals is component specific to a certain extent but also that the development of some empathy components in ASC individuals is relatively unstable. Specifically, although the development of some empathy components in ASC individuals showed a similar age trend as the age trend of typically developing individuals, the increase and decrease in empathy components are more obvious in ASC individuals than in typically developing individuals. The age-increasing effect of the T-EA advantages in ASC individuals may benefit from the age-aggravating effect of T-CE impairment.

## Prospects and Limitations

In this study, a meta-analysis was used to investigate the impairment of specific empathy components in individuals with autism and the moderating effects of culture, gender, and age from a more microscopic perspective. Finally, we found that the empathy impairment of ASC individuals is component specific. That is, the trait-cognitive empathy, trait-empathic concern, state-cognitive empathy, and state-empathic concern are impaired, whereas state-empathic accuracy remains intact, and trait-empathic accuracy is superior to the trait-empathic accuracy found in neurotypical individuals. Moreover, the impairment of different empathy components in ASC individuals is also regulated by gender and age to some extent. The practical significance of these findings lies in the fact that the results provide not only a reasonable and scientific explanation for the controversy regarding the definition, structure of empathy and empathy impairment of ASC individuals to a certain extent but also insights into clinical interventions regarding the impairment of social communication in autistic individuals. Specifically, based on respect for the natural differences of sex and age in the empathy impairment of ASC individuals, clinical interventions for empathy impairment in ASC individuals should draw lessons from the four methods of behavioral skill training (instruction, modeling, practice, and feedback; Emily and Malouff, 2016). Meanwhile, more attention should be focused on the intervention of state-cognitive empathy and state-empathic concern in ASC individuals to drive the natural ease of the impairment of other empathy components. It is worth noting that, due to inherent differences among the empathy components, formulating the training content according to the specific connotations of state-cognitive empathy and state-empathic concern would be an effective strategy.

Of course, there are still some limitations to our study. First, due to the limited number of studies on Eastern culture and with female subjects, the specific moderating effects of culture and gender on T-CE, T-EA, S-EC, and S-EA impairment in ASC individuals were not investigated. Therefore, studies on the impairment of the different empathy components in ASC individuals based on Eastern collectivistic culture samples and female samples should be pursued in the future. Second, the influence of culture, gender, and age on the empathy impairment of ASC individuals is not synchronized at the trait and state levels, and the reasons for this are not clear. Future studies should further clarify the interaction mechanism among the three components and the two levels of empathy and take this as a breakthrough point to further examine the cross-culture, cross-gender, and cross-age stability of different components of empathy. Third, to investigate the moderating effect of gender on the empathy impairment of ASC individuals, we divided the specific research into two categories, predominantly male vs. predominantly female, and then conducted an analysis on the moderating effect of gender; it is not clear how effective this approach was. Therefore, in future studies, the empathy impairment of ASC individuals of different genders should be explored separately, or the empathy impairment of ASC individuals of a certain gender should be explored independently to achieve a purer demonstration of the moderating role of gender in ASC individual empathy impairment.

In addition, the specific empathy impairment of ASC individuals may be influenced by other factors, such as intelligence (Bertollo and Yerys, 2019), cognitive control (Hughes, 2001; Bertollo and Yerys, 2019), action anticipation (Sinha et al., 2014; Krogh-Jespersen et al., 2018), and implicit learning (Lieberman, 2000; Foti et al., 2015), among others. Especially relevant may be the empathy impairment of ASC individuals at the state level due to the situational dependence of state empathy itself (Kennedy and Adolphs, 2012; Chen and Liu, 2016; Powell and Roberts, 2017). Consequently, it is more vulnerable to the influence of experimental materials. However, due to the incomplete and inconsistent information provided in previous studies, or the unbalanced use of different experimental materials, it is difficult to form effective coding; therefore, we did not pursue further analysis of these factors. Nevertheless, it also implies that the regulatory factors for the specific empathy impairment in ASC individuals are still worthy of further investigation.

## Conclusions

1. The empathy impairment of ASC individuals is component specific; that is, ASC individuals have impairment in T-CE, T-EC, S-CE, and S-EC, whereas the S-EA and T-EA components are intact or better than the S-EA and T-EA of neurotypical individuals.
2. Gender moderates the impairment of T-EC, T-EA, and S-CE in ASC individuals. Age moderates the impairment of T-CE, T-EA, S-EC, and S-EA in ASC individuals.

## Data Availability

All datasets analyzed for this study are included in the manuscript and the supplementary files.

## Author Contributions

YS conceived of the study, performed the statistical analysis, and drafted the manuscript. TN participated in the collection and selection of the literature. WS conceived of the study and helped draft the manuscript. XZ and YY participated in the literature coding and some data collection. All authors read and approved the final manuscript.

### Conflict of Interest Statement

The authors declare that the research was conducted in the absence of any commercial or financial relationships that could be construed as a potential conflict of interest.
